# The expanding array of HIV broadly neutralizing antibodies

**DOI:** 10.1186/s12977-018-0453-y

**Published:** 2018-10-16

**Authors:** Laura E. McCoy

**Affiliations:** Division of Infection and Immunity, University College London, London, UK

**Keywords:** Antibody, Neutralization, HIV, Immunity, Infection, Epitope

## Abstract

A large array of broadly neutralizing antibodies (bnAbs) against HIV have been isolated and described, particularly in the last decade. This continually expanding array of bnAbs has crucially led to the identification of novel epitopes on the HIV envelope protein via which antibodies can block a broad range of HIV strains. Moreover, these studies have produced high-resolution understanding of these sites of vulnerability on the envelope protein. They have also clarified the mechanisms of action of bnAbs and provided detailed descriptions of B cell ontogenies from which they arise. However, it is still not possible to predict which HIV-infected individuals will go onto develop breath nor is it possible to induce neutralization breadth by immunization in humans. This review aims to discuss the major insights gained so far and also to evaluate the requirement to continue isolating and characterizing new bnAbs. While new epitopes may remain to be uncovered, a clearer probable benefit of further bnAb characterization is a greater understanding of key decision points in bnAb development within the anti-HIV immune response. This in turn may lead to new insights into how to trigger bnAbs by immunization and more clearly define the challenges to using bnAbs as therapeutic agents.

## Background

HIV infection remains a major global health challenge but to date, it has not been possible to induce protective immunity against this virus by vaccination. This is different to the situation with other viral pathogens, such as measles, where the immune response triggered by vaccines, specifically the antibodies induced provide complete protection from disease. In contrast, antibodies induced by both natural HIV infection and vaccine candidates generally are not of sufficient quality to protect from infection. This is largely because they are highly specific for the distinct strain of HIV to which the individual was exposed, or even specific for a just a small proportion of the intra-patient quasi-species in the case of an infected individual. Furthermore, the viral protein they bind, the envelope glycoprotein (Env), is expressed at a low level on the viral surface in a unstable conformational state. Therefore, it is challenging for the immune system to produce effective antibodies against HIV most antibodies. Thus, they cannot prevent infection by circulating HIV strains in the general population. However, in a rare subset of HIV-infected individuals antibodies arise that are able to recognize and block an extremely wide array of HIV strains. These are known as broadly neutralizing antibodies (bnAbs) and are so highly functional due to a combination of extensive somatic hypermutation and unusual structural features, notably very long complementarity determining region (CDR3) loops. Since 2009 the identification of a huge number of bnAbs has provided new impetus for HIV vaccine research. This review will explore what has been learnt from this renaissance in HIV antibody research, what remains to be understood and crucially whether we need to continue to isolate HIV bnAbs given the exponential discovery rate of these remarkable antibodies over the last decade.

## Why study bnAbs against HIV?

Shortly after the identification of HIV as the causative agent of AIDS it became clear that antibody responses in infected patients were mainly limited to neutralizing only HIV strains closely related to the infecting virus [1–3]. This raised the idea that the induction of bnAbs would be a necessary step in the development of a protective HIV vaccine. However, some years elapsed before the description of the first monoclonal antibody (mAb) with the capability to neutralize divergent HIV strains [4]. This discovery demonstrated that the human immune system could indeed produce such highly functional antibodies. This mAb, b12, was followed by a handful of additional bnAbs (4E10, 2F5, 2G12). These discoveries were crucial conceptually, in that they demonstrated clearly that antibodies can block in vitro infection by a wide-range of HIV strains and are not always limited by strain-dependent differences. Moreover, the isolation of these bnAbs facilitated landmark in vivo experiments which showed that it is possible to protect animals from infection via both high-dose and repeated low-dose challenge [5–10]. This demonstrated the principle that the presence of bnAbs at a systemic level can prevent infection and provided an immunological benchmark to aim for during vaccination studies. The isolation of additional bnAbs in the last decade has confirmed the potential of passive transfer of these antibodies. Many of the new bnAbs have significantly improved potency and this is reflected by the smaller doses required to protect from infection [11] and that protection can be achieved even with bnAbs that result in incomplete neutralization at low concentrations in vitro [12]. Moreover, recently it has been seen that a single dose of a bnAb can protect from repeated infectious challenge [13] and that dosing after infection can result in a degree of virological control [14, 120]. Thus, the stage is now set for the adaption of bnAbs for use as therapeutic/prophylactic agents in humans. In turn, this progress raises the central question of this review: are there now enough HIV bnAbs? To address this, it is first necessary to consider what information has been learnt so far from studying bnAbs and what insights this has provided.

**Fig. 1:**
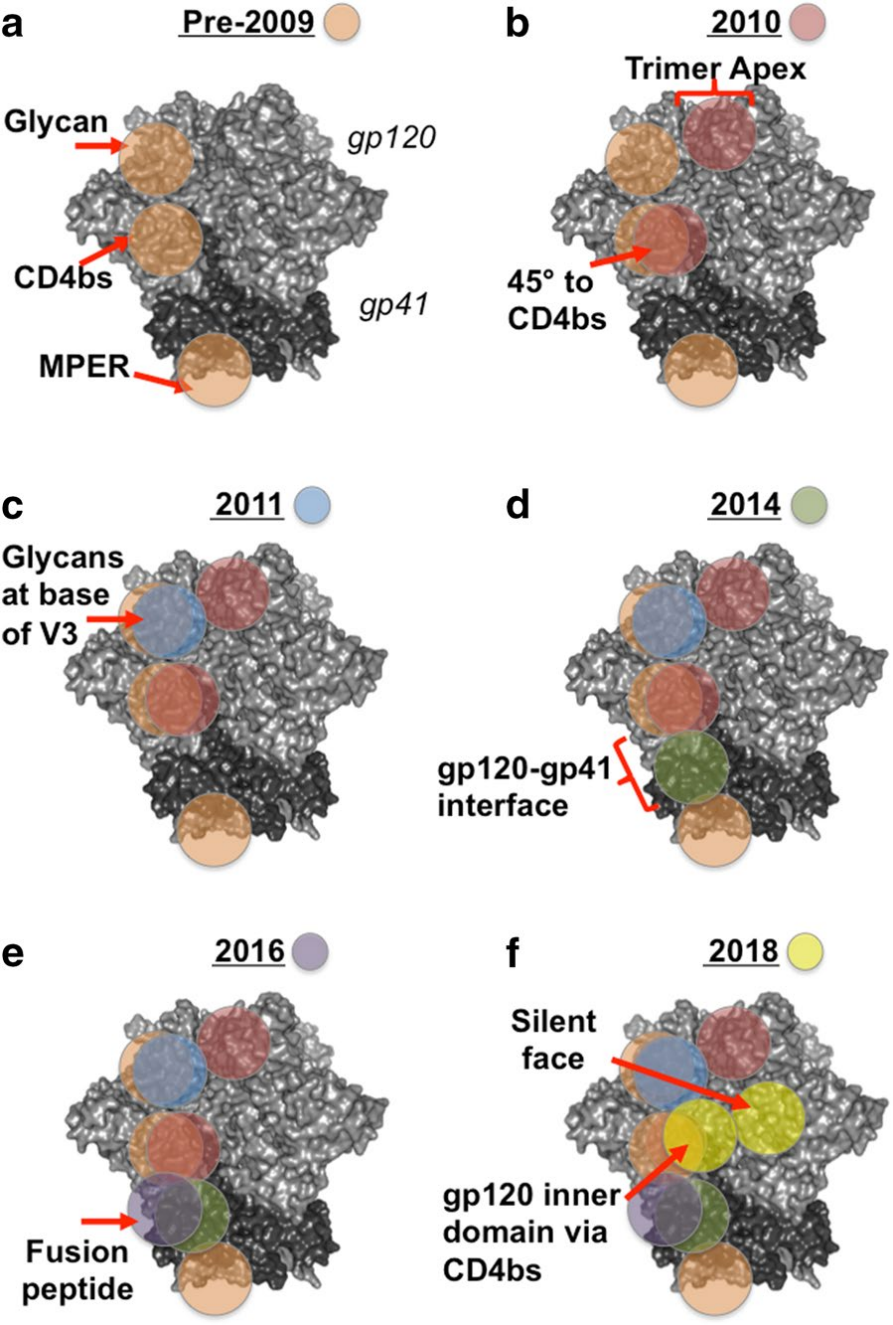
Continual advances in identifying bnAb epitopes on HIV Env following the identification of new bnAbs. Each panel represents a key advance in the identification of new epitopes/refinement of epitopes bound by bnAbs. The Env figure is adapted from the crystal structure of the BG505 SOSIP.664 trimer (PDB: 5cez), gp120 is coloured light grey, gp41 is coloured dark grey. Approximate epitope locations are indicated by red arrows/lines and circles are color-coded for each year as shown in the key given in each panel heading. Epitopes are highlighted only once per protomer. **a** Pre 2009 knowledge of CD4bs, glycan and MPER epitopes gained from studying predominantly by b12, 2G12, 2F5 and 4E10 respectively. **b** By 2010 the trimer apex epitope had been described following discovery of PG9/16 in 2009 and the importance of angle of approach to the CD4bs highlighted by the discovery of VRC01 in 2010. **c** The glycan patch epitope was redefined as supersite of vulnerability by the isolation of the PGT121 and 128 families of bnAbs in 2011. **d** From 2014 onwards the discovery of additional bnAbs, including PGT151, 35O22 and 8ANC195, revealed a new area of bnAbs which span the gp120–gp41 interface. **e** In 2016 subunit interface targeting antibodies were found that also bind the gp41 fusion peptide, VRC34 and ACS202. **f** 2018 saw the description of bnAbs binding the highly glycosylated “silent” face of gp120 and targeting CD4bs via novel contacts with the gp120 inner domain after bypassing the Phe43 cavity

The most fundamental information gained from the studying of bnAbs since the isolation of b12 has been the definition of bnAb binding sites on HIV Env. Specifically in terms of the limits they impose on antibody binding which render them challenging targets to hit by vaccination. The five key bnAb epitopes are the CD4 host receptor-binding site (CD4bs), the high mannose patch, the Env trimer apex, the membrane proximal region (MPER) and the subunit interface region between the gp120 and gp41 subunits of Env (Fig. 1). Of these, the CD4bs, MPER and high mannose patch were identified by bnAbs first described in the 1990s/early 2000s. However, the exponential growth in bnAb identification since 2009 has provided great insight into the biology of HIV Env, including the definition of two new major bnAb binding sites (the apex and interface) as illustrated in Fig. 1. Thus, the expanding array of HIV bnAbs continue to re-define our molecular understanding of the neutralizing epitopes on Env and the challenges associated to inducing bnAbs by vaccination.

## Insights into HIV Env: CD4bs

A logical mode of action for an HIV bnAb is to interfere with the binding of HIV, via Env, to the human host receptor protein CD4. Not only because it is a crucial step in viral entry but also because the high variability of HIV Env is constrained in the CD4bs as certain features must be conserved to maintain the interaction with the host receptor. Indeed, the earliest described bnAb, b12, binds to the CD4bs of Env [4]. However, the viral entry process facilitated by the interaction between Env and CD4 is complex and involves multiple conformational changes within the viral protein. This became apparent early on from studies comparing b12 with other mAbs that target the CD4bs but are not able to neutralize primary HIV isolates in vitro [15] nor provide protection from infection after passive transfer [16]. Studies with the gp120 subunit of Env revealed that the molecular contacts of non-neutralizing CD4bs antibodies differ from those of neutralizing CD4bs antibodies [17]. Subsequently cryo-electron microscopy has confirmed that the non-neutralizing CD4bs antibodies bind to an opened-up form of the Env gp120–gp41 trimer not the tightly-associated trimer which is needed to engage CD4 and enable infection [18]. Understanding of the CD4bs bnAb epitope has accelerated rapidly since the description of the second CD4bs bnAb VRC01 in 2010 [19]. This bnAb and its clonal variants disproved the notion that the best possible CD4bs bnAb would closely mimic human CD4. Instead of mimicking CD4, VRC01, which neutralizes ~80% of HIV strains as opposed to the ~ 40% neutralized by b12, binds at a 45 °C angle relative to the orientation of CD4 binding to Env [19]. This preferred angle of approach for CD4bs bnAbs has proven to be a general requirement, not only in the vast family of VRC01 variants [20] but also in genetically distinct bnAbs [21]. A precise binding angle is needed to enable the bulky fab fragment of the bnAb to enter the CD4bs, which is recessed in what can be termed a canyon on the surface of Env. Recessed host receptor binding sites are a frequent feature of pathogens, for example polio virus, as this can limit recognition by the host antibody responses [22, 23]. That HIV utilizes a similar mechanism was predictable from early structural studies of CD4 binding to Env. However, it was the isolation of a large number of CD4bs BnAbs, all binding at similar angles, that proved this was a major challenge for inducing antibodies against this site. Furthermore, in-depth study of CD4bs bnAbs has defined an epitope signature of molecular contacts that this class of bnAbs use to bind Env [24] Such extensive characterization of CD4bs bnAbs has also revealed that HIV adds to the geometric obstacle posed by the location of the CD4bs by presenting a high level of amino acid diversity around the entrance to the canyon. The blocking effect this achieves is amplified by post-translational modifications, namely N-linked glycosylation. Specifically, the presence of N-linked glycans close to the CD4bs can be highly obstructive, and there is some evidence they limit the binding of CD4bs bnAb precursors, thus impeding the development of mature CD4bs bnAbs [25]. Despite the intrinsic challenge in targeting the CD4bs many bnAbs against this site continue to arise during natural infection. Recently, a CD4bs bnAb, N6, was identified that is even more potent and described as displaying near-pan neutralization breadth [26] and has been found to suppress plasma viral loads in a non-human primate model [14]. Moreover, new methods for bnAb identification continue to emerge as illustrated by the description of another CD4bs bnAb N49P7 [27]. This antibody was identified directly from plasma using proteomics and antibody lineage analysis. Importantly, N49P7 binds the CD4bs in a new fashion, bypassing the Phe43 cavity and instead contacting the inner domain of gp120 [27].

## Insights into HIV Env: high mannose patch

One of the most unusual and intriguing early bnAbs was 2G12 [28]. This unusual domain-swapped antibody directly recognizes N-linked glycans close to the third variable loop (V3) of gp120. The sugar moieties covering Env are unlike bacterial polysaccharides, which are well-recognized by the human antibody response and form the basis for some preventative vaccines. The N-linked polysaccharides which comprise approximately 45% of the total mass of Env are fundamentally human in origin. This is because Env is produced in host cells and undergoes post-translation modification with human glycan processing enzymes. Therefore, these structures are largely tolerated by the immune system and minimally immunogenic. This explains the observation that intra-patient viral quasi species gain more potential N-linked glycan sites (PNGS) over time and that this is associated with a loss of serum neutralization activity, as the neutralization epitopes are hidden by the extra glycans [29, 30]. However, the joint presentation of N-linked glycans with viral protein at an unusually high density on certain parts of Env can be recognized by human antibodies. Until 2011 the only well-defined glycan-specific bnAb was 2G12 and attempts to re-elicit such specificities had induced glycan-specific antibodies but they were not able to neutralize HIV [31]. The description of the PGT121 and PGT128 bnAb families in 2011 demonstrated that reactivity with this dense patch of mainly high mannose glycans is not solely possible with a domain swapped antibody [32]. On the contrary, this specificity is one of the most commonly found in patients with bnAb activity in their sera [33, 34]. This is a striking observation given the minimally-immunogenic nature of N-linked glycans in humans, and the observations that HIV uses the hosts’ sugars to create a glycan shield to hide behind [29]. It suggests that in chronic HIV infection, the pressure on the humoral immune system to halt the virus is strong enough to make even N-linked host glycans a viable target. On a molecular and structural level, the study of the PGT121 and 128 families revealed a particular glycan, which alternates between position N332 or N334 within Env, was a key lynch pin for binding and neutralization by this class of bnAb [32]. Isolation of additional clonal variants and unrelated bnAbs targeting the same site led to structural comparison studies which highlighted the divergent modes of recognition and angles of approach possible for these BnAbs [35, 36], which is a stark contrast to CD4bs bnAbs. This high mannose site has thus been termed a supersite of vulnerability and recent work has shown that separate bnAb families against this site can arise within the same individual [37]. That there are so many structural and genetically diverse ways for antibodies to target the high mannose site has led to renewed efforts to design vaccine candidates to induce such antibodies [38, 39]. Moreover, high mannose patch specific bnAbs including PGT121 and 10-1074, have shown great promise in passive transfer studies both in regard to preventing infection at low doses [11] and controlling established infection [14, 120]. Notably, data in these studies do highlight the risk of escape mutations if bnAbs are used as monotherapy. While this will undoubtedly hold true for all specificities, intensive study of individual glycan-patch specific bnAbs has shown that if the loss of the N332/N334 glycan does not enable escape, HIV will escape sometime by unusual mutations such as the introduction of disulfide bonds [40, 41]. In addition to highlighting how the virus can escape from antibodies, such detailed studies of individual high-mannose patch bnAbs have also suggested reasons why mechanistically this particular part of the glycan shield is a good bnAb epitope. Namely, that it includes a motif that is associated to CCR5 co-receptor binding [42] and thus it is a key part of the viral entry process.

## Insights into HIV Env: MPER

The MPER is another example of a bnAb epitope that was originally described before the recent wave of HIV bnAb isolation. However, since 2009 only one additional MPER bnAb, 10E8, has been studied in great detail [43]. Regardless, characterization of 10E8, alongside new studies with previously described MPER bnAbs, have provided valuable insight. Namely, that there have been refinements in our molecular [44, 45] and structural understanding of how this epitope is recognized [46, 47] and the particular obstacles faced when attempting to elicit MPER bnAbs following vaccination. The original MPER bnAbs, 2F5 and 4E10, bind to an overlapping linear epitope in the gp41 subunit. The movement of gp41 is a key step during viral fusion, thus as per the CD4bs, the location of MPER itself suggests why these antibodies can effectively prevent infection. Moreover, recent work has shown that 10E8 can actually destabilize the Env trimer which the authors describe as a novel mechanism of neutralization [48]. MPER is highly conserved across HIV strains and as a result this class of bnAbs have the potential for great breadth, as seen with 4E10 [49] and also potency, with both attributes combined in 10E8 [43]. This new MPER bnAb has also been shown to protect non-human primates from infectious challenge [50]. All of this makes MPER an attractive target for vaccine design, particularly as a linear epitope is easier to manipulate than a conformational epitope and many different approaches have been explored to present the MPER peptide to the immune system [51]. However, detailed characterization of the binding of 2F5, 4E10, and more recently 10E8, has shown that the MPER bnAb epitope is complicated by its proximity to the viral membrane and that neutralization is achieved in part via lipid binding as reviewed in [52]. This suggests that the MPER epitope needs to be presented in the context of lipid to induce neutralizing antibodies. The flipside to this is that lipid-reactive antibodies, like N-glycan reactive antibodies, are essentially binding to a host component. Indeed, it was initially shown that this potential for autoreactivity negatively regulates the development of 4E10-expressing B cells in transgenic knock in mice [53]. More recently, deletion of 2F5-expressing B cells has been observed in knock-in mice [54]. Macaques immunized with a 2F5-tailored immunogen did make 2F5-like B cell clonal lineages but with insufficient affinity to neutralize virus [54]. The authors suggest this is because gp41 bnAbs are limited by immune tolerance mechanisms against lipid binding. The authors further propose that vaccination will require intervention to overcome immunological tolerance [54] as reviewed in [55]. Notably MPER bnAbs are found during natural infection, despite the blocks to the development of these bnAbs seen thus far in animal models. This contrast in MPER-specific B cell fate is most starkly seen in a recent study showing that B cells producing MPER bnAbs can differentiate into both peripheral memory B cells and long-lived bone marrow plasma cells in vivo [56]. Moreover, in this particular HIV-positive individual, the inferred common ancestor of the entire MPER bnAb family was found to be autoreactive, which supports the argument that tolerance needs to be compromised for MPER bnAb development [56]. However, a separate study showed vaccination could induce long-lived bone marrow plasma cells that produce MPER antibodies that aren’t autoreactive, although notably they were also not bnAbs [57]. Thus, there remains debate about the limits imposed by immunological tolerance in the development of bnAbs, particularly against MPER.

## Insights into HIV Env: trimer apex

Arguably, the identification of new epitopes has been the most significant output from the characterization of HIV bnAbs over the last decade. The first new bnAb epitope described was that bound by PG9 and PG16, a pair of somatic variant antibodies, which were the first in the new wave of bnAbs [58]. Crucially the identification of novel epitopes was made possible by using an unbiased selection method as reviewed in [59]. The landmark study by Walker et al. [58] showed that these antibodies recognized a highly conserved epitope centered on an N-linked glycan at N160, which is preferentially expressed on trimeric Env and spans conserved regions of first and second variable loops (V1/V2) of the gp120 subunit. Structural studies revealed that PG9/16 bind in a heavy chain dominated fashion, using a long third heavy chain complementarity determining region (CDRH3) in what was termed a “hammerhead” structure to bind to the V1/V2 at the very top of the Env trimer where the three gp120 subunits meet to form the trimer apex [60]. Later work redefined the precise molecular requirements of the apex class of bnAbs, including PG9/16 alongside other bnAbs, and the contribution to the paratope made by bnAb framework regions [61]. Furthermore, additional structural studies on the PGT145 apex bnAb [58] confirmed previous work on the trimeric nature of this epitope by demonstrating the CDRH3 penetrates between glycans at the trimer threefold axis, to contact peptide residues from all three Env protomers [62]. In addition, a novel apex bnAb, BG1, was observed to bind asymmetrically to Env using a compact CDRH3 rather than a hammerhead structure [63]. Thus, this bnAb binds in a 2:1 ratio to Env trimer, rather than 1:1 as per classical apex bnAbs such as PG9 [63].

Similar to the high-mannose patch bnAbs, apex bnAbs not only successfully navigate around the glycan shield of Env but also bind directly to N-linked glycans. The original description of PG9/16 highlighted the crucial importance of the N160 glycan in particular for this class of bnAbs [58]. More recently, in depth analysis of precursor antibodies of another apex bnAb, VRC26, have shown a preference for sialic acid-bearing glycans [64]. This work also highlighted that binding to these glycans served as an “anchor” for the nascent bnAb, regardless of amino acid variation in the epitope. Thus rendering the antibody lineage resistant to complete neutralization escape and allowing the eventual development of breadth [64]. As discussed above, the utilization of N-linked glycans by apex and high-mannose patch bnAbs is at odds with the observations on the immunosuppressive nature of Env glycans. Namely, that “holes” in the glycan shield are highly susceptible targets for immunization-induced neutralization [65] and that adding glycans to Env hides neutralizing epitopes from the immune system [29]. So the observation that these two classes of bnAbs recognize these generally non-immunogenic structures suggests that the regulation of the cells producing them has been altered in some way, perhaps also involving alterations in tolerance as suggested for MPER bnAbs. An alternative explanation for N-glycan reactivity is that the particular sugars eliciting bnAbs are altered in some way in comparison to glycans on host proteins. This idea is suggested by work showing very high avidity binding of PG9 to synthetic hybrid glycans, which led the authors to propose that these unusual sugars may have been the original ligand for the PG9 bnAb family [66].

## Insights into HIV Env: gp120–gp41 interface

The trimer apex is not the only new bnAb epitope to have been identified by the isolation of new bnAbs. The isolation of PGT151 [67] defined the interface between the gp120 and gp41 subunits as an area targeted by bnAbs. Notably, this novel specificity was also identified by the unbiased selection of a bnAb for neutralization activity rather than using proteins presenting known bnAb epitopes as reviewed in [59]. One of the unique features of this first-in-class interface bnAb is its requirement for complete cleavage of the subunits prior to trimer assembly [68]. This meant that much of the original characterization was performed with cell-surface Env derived from the JRFL strain as available soluble Env proteins were not adequate mimics of the interface site [67]. As highlighted above, rearrangement of the Env subunits is a crucial part of the viral entry process and so a logical target for a neutralizing antibody. Moreover, there is a level of conservation in this region across viral strains, due to the need to maintain the correct oligomeric structure. However, the subunit interface had not previously been considered as a bnAb target, in part due to the predominant experimental use of separate gp120 and gp41 proteins. This technical limitation has been overcome in the last decade by the development of native-like Env trimers [69–72]. The development of new trimers and the characterization of interface bnAbs have been mutually beneficial, with PGT151 in particular being of great use to exclusively purify properly cleaved trimers by affinity chromatography [73]. The same holds true for the apex bnAbs, which can be used to select for tightly folded recombinant trimer [73–75]. Another similarity is that PGT151 also recognizes N-linked glycans as part of its epitope, although in this case it requires the loss of two separate glycans to destroy the epitope and prevent neutralization [67].

Simultaneous with the description of PGT151 additional bnAbs were found to target the subunit interface region of vulnerability but each via distinctive Env contact sites. Notably, many were also identified by a neutralization based selection method, including 35O22 [76]. Interestingly, 35O22 is derived from the same source individual as the MPER bnAb 10E8. 35O22 like PGT151 is trimer specific, but it is different in that can bind both cleaved and uncleaved forms of Env. Another similarity between these interface bnAbs is that the removal of specific glycans from Env decreases their neutralization activity. Namely, N88, N230, N241 and N625 in the case of 35O22 [76]. Furthermore, for particular viruses, both bnAbs can achieve only 50–80% neutralization even at very high concentration of antibody. This incomplete neutralization phenomenon has been observed with all classes of bnAbs and is due to resistance in a fraction of the virus population arising due to glycan and possibly conformational heterogeneity [77, 78]. Thus, study of interface bnAbs has highlighted the extensive post-translational variation in any given population of HIV virions and the challenge this poses to preventing infection. 35O22 also gave rise to greater insight into the fusion process required for HIV entry. Previously, it was shown the MPER bnAbs bind more efficiently after the conformational changes induced by CD4 engagement [79]. 35O22 was also observed to bind poorly to membrane-bound Env prior to CD4 engagement and to prefer an early intermediate conformation during fusion. The authors speculated that this could be because the Env is raised within the viral membrane at the start of fusion that leads to greater exposure of the 35O22 epitope [76]. Notably, another of the interface bnAbs, 8ANC195 [80], actually alters the conformation of Env by inducing a partially closed form of the CD4-bound trimer [81]. Thus, characterization of 8ANC195, confirmed that Env exhibits a high level of conformational heterogeneity and revealed a previously unseen conformation. It is important to note that this is the highly diverse class of interface bnAbs, which bind a common area on the trimer rather than closely overlapping epitopes as is the case with the other classes discussed above. For example, two new bnAbs (ACS202 and VRC34) have been described which target the interface area but actually crucially interact with the Env fusion peptide [82, 83]. ACS202 and VRC34 bnAbs also exhibit common interface bnAb features such as a requirement for trimeric Env and including N-linked glycans within their epitope. Recently another interface bnAb, named CAP248-2B, similar to PGT151, was identified which, like PGT151, also partly binds via the fusion peptide [84]. Intriguingly, mutations that abrogate the neutralization of CAP248-2B actually increase the susceptibility of the virus to neutralization by MPER and other interface bnAbs [84]. Thus highlighting a reoccurring theme in the isolation and characterization bnAbs, namely that combining a range of specificities [85] has great potential for therapeutic applications and vaccine development.

## Insights into HIV Env: all bnAb epitopes

Combining the knowledge generated by the extensive array of bnAbs identified to date also provides important insights into HIV Env biology and new tools with which to evaluate immune responses against HIV. This is most clearly exemplified by the generation and validation of highly defined panels of pseudoviruses and epitope specific mutant viruses [86–88]. These tools enable quantification of the level of neutralization breadth across large cohorts and rapid detection of bnAb specificities within polyclonal serum samples [89]. These mapping tools have the potential to not only identify a larger number of individuals with broadly neutralizing sera but also to facilitate detection of low levels of activity or similar specificities in immunization studies. This in turn may help in the stepwise development of HIV Env vaccine candidates. Moreover, the knowledge garnered from the extensive study of bnAbs allows a more exacting investigation of host-virus specific immune responses during chronic infection. This in turn may be able to support work towards personalized immunotherapeutic approaches for HIV. Crucially the study of HIV bnAbs over the last decade has comprised a combination of epitope-focused and open-ended antibody discovery. This has facilitated highly detailed studies of how the particular bnAb classes function alongside the identification of new bnAb epitopes leading to insights into the fundamental biology of Env and the HIV entry process.

## Why continue to study bnAbs against HIV?

A reoccurring theme throughout the study of HIV bnAbs during the last 10 years is that new epitopes are often discovered, despite earlier comprehensive studies [90, 91]. This is exemplified by the identification of the interface bnAbs [67, 76, 80] and the more recent description of interface bnAbs that use the fusion peptide to neutralize virus [82, 83]. One of the most recent example of the identification of new epitopes is the description of a single antibody, VRC-PG05, which recognizes a region on gp120 known as the silent face, comprising a dense N-linked glycan patch thought previously to be resistant to neutralizing antibodies [92]. VRC-PG05 binds directly to this “silent” glycan patch to neutralizes around 30% of viruses tested [93]. Intriguingly, electron microscopy studies suggest that only two copies of the antibody bind a single trimer, thus there is space for one CD4 molecule also to bind, leading the authors to state that VRC-PG05 does not prevent CD4 binding to Env. Instead they propose that this antibody neutralizes viruses by impeding the conformational changes that allow CD4 to bind all three of its binding sites on Env and promote viral fusion [93].

In addition to the identification of new bnAb epitopes, continuing to isolate and characterize bnAbs can highlight the differences between bnAbs in each class [21]. This in turn can lead to greater understanding of why it is challenging to induce such antibodies by immunization. In particular, the study of bnAb families or lineage studies have been highly informative. Early work in this area highlighted that predicted unmutated common ancestors (UCA) or inferred germ line (iGL) versions of most HIV bnAbs do not bind to Env with higher affinity [94, 95] and has led to concerted efforts to improve Env interactions with putative bnAb precursors. UCA/iGL binding to Env has only been observed in two distinct situations. Firstly, where precise recapitulation of the eliciting viral strain is possible [88, 96]. Secondly, where a large part of the paratope is formed by an exceptionally long CDRH3 region and thus present prior to affinity maturation [61]. Importantly, these long CDRH3 antibodies are rare in human B cell repertoires posing a challenge for expansion upon immunization [97]. Characterization of early members of bnAb families has also been highly informative. Particularly when neutralization breadth is observed with antibodies of a similar level of affinity maturation (~ 10% somatic hypermutation) to those commonly elicited by vaccination [36]. Furthermore, combining bnAb lineage studies with viral phylogenetics has begun to show how epitope diversification may lead to the development of breath. Notably in one case due to partial viral neutralization escape over a prolonged period resulting in an expanded time window for bnAb maturation to occur [98]. Moreover, studying the ontology of bnAb families has illustrated that these rare antibodies are part of a larger Env-specific antibody repertoire within HIV-positive individuals. Firstly, there was the description of a helper lineage that exerted selection pressure on the viral quasi species to drive it to form the epitope for an emerging bnAb family [99]. More recently, it has been reported that strain-specific neutralizing antibodies and bnAbs can function in parallel to limit escape by viral mutation and by doing so enhance the exposure of a bnAb epitope [100]. However, to date there has been minimal investigation role of non-neutralizing antibodies in bnAb-producing individuals, despite recent observations that such antibodies can alter HIV infection in humanized mice [101].

Another important reason to continue searching and characterizing bnAbs is that this work advances the ability of the field to analyze post-immunization responses. Limited progress has been made in inducing bnAbs by immunization, except in transgenic mice and animals with aberrantly structured antibodies [38, 102–104]. However, the panel of bnAbs available have allowed refinement of new immunogens to increase bnAb affinity and limit binding to non-neutralizing epitopes [70, 71, 74, 105]. Moreover, comparing the binding of neutralizing mAbs isolated post-immunization to those of bnAbs has provided much greater understanding of why breadth has not been induced [65]. Furthermore, the wealth of knowledge generated on different bnAbs and their family members has also enabled the application of computational modeling approaches to the problems of how to induce these antibodies. This has recently been attempted in terms of the dynamics of germinal centers [106], the fitness landscape of Env [107] and the mutability of antibodies [108]. However, importantly, predictions based on these computational approaches require experimental validation. Already this has been attempted with earlier theoretical work focused on understanding the likelihood of bnAb precursor activation and clonal expansion. This was achieved by altering the frequency of bnAb precursors present in a transgenic mouse model [109]. Strikingly, this study revealed that both a threshold frequency and affinity are required for bnAb precursors to expand during an in vivo immunization [109].

Studying HIV bnAbs has also contributed to greater understanding of the basic rules underpinning the development of antibodies and concurrent immunology. For example, isolation of bnAbs has highlighted that antibodies raised in infants can be highly functional without extensive hypermutation [110]. In addition, analysis of the B cell repertoires in bnAb-producing individuals has highlighted that different bnAb family members are found in altered proportions in peripheral and bone marrow compartments [56]. Furthermore, consideration of the multiple bnAbs isolated to date, and in particular bnAb B cell ontogeny studies have led to speculation about what the limits to B cell affinity maturation are and whether it is even possible to drive such extensive mutation by vaccination [111]. This has coincided with increased investigation into the virological and inflammatory profiles associated to the development of HIV bnAb-like activity [112, 113] and the complex nature of the relationship between escape virus populations and antibodies in vivo [114, 115].

## How can we use bnAbs against HIV?

An alternative consideration to what we can learn from bnAbs is how can we practically use the expanding array of bnAbs? That bnAbs can protect animals from experimental challenge is one of the main pieces of evidence, which originally suggested that vaccines that can induce such antibodies would be protective. However, given the difficulties of inducing bnAbs by immunization, a reasonable short-cut for many is to deliver well characterized bnAbs directly as either therapy or in a prophylactic setting. Both the use of passive antibody infusions and vectored-antibody prophylaxis are being pursued with HIV bnAbs as reviewed extensively elsewhere [116, 117]. These approaches are evaluating the usefulness not only of naturally occurring bnAbs but also composite forms including bi- and tri-specific engineered antibodies that target multiple bnAb epitopes. Recent key bnAb studies in animal models and HIV-positive humans (Table 1) have highlighted the potential for bnAbs to protect from infection [118], to delay viral re-bound following anti-retroviral treatment cessation [119], to maintain viral supression [120] and to act as an adjunct to host immune-mediated control of virus [121]. Importantly, how and when these approaches can be integrated with current standards of care for patients remains to be clarified. There is growing interest in utilizing bnAbs in immunotherapeutic interventions aimed at curing infection as reviewed elsewhere [122, 123]. However, a recent observation, that super infection occurred in the presence of passively infused bnAb in an animal model [124], highlights the need for caution. Moreover, this work illustrates the need for a greater understanding of how bnAbs function and crucially how they impact ongoing infection in the infected individuals in which they develop. In turn, this supports the continued isolation and study of bnAbs.

**Table 1:** Selection of recent protection/treatment studies using bnAbs

BnAb delivery	Epitope	System	References
Adeno-associated virus (AAV) vectored PGT121	High mannose patch	Humanized mice	[118]
Passive infusion of 3BNC117 and 10-1074	CD4bs High mannose patch	Non-human primates	[121]
Passive infusion of a Tri-specific mix	CD4bs MPER Apex	Non-human primates	[125]
Passive infusion of VRC01	CD4bs	Humans (phase I)	[126]
Passive infusion of VRC01	CD4bs	Humans phase 1	[127]
Passive infusion of VRC01	CD4bs	Humans phase 1	[119]
Passive infusion of 3BNC117	CD4bs	Humans phase 2a	[128]
Passive infusion of 3BNC117 and 10-1074	CD4bs High mannose patch	Humans phase 1b	[120]

In conclusion, the study of HIV bnAbs since 2009 has generated a huge body of knowlege as to how antibodies can overcome the inherent obstacles in targeting a highly variable pathogen with a conformational variable surface protein, such as HIV. Primarily, this is achieved by bnAbs binding to regions the virus cannot alter without drastically limiting itself. These regions include not only highly conserved amino acids at the receptor binding site but also to contacts needed to maintain envelope protein trimerisation. Moreover, bnAbs directly bind to the N-linked glycans that cover Env and actually facilitate evasion of less effective antibodies. The bnAbs identified are now being advanced to potential clinical applications but there remains much to learn by continuing to search for new bnAbs and in studying the processes by which they develop. Particularly, there is a need to understand more clearly the development of BnAbs in the context of the wider HIV specific antibody repertoire in the patients that produce them. It will be important to investigate the relative abundance of bnAbs within the antibody repertoire of the host, and the impact this has on their development in vivo. This in turn will suggest ways we can attempt to mimic bnAb generation by vaccination. Moreover, continued understanding of the varying ways in which bnAbs can bind their epitopes, in particular greater knowledge of any mechanistic limitations, will provide much needed insight into the therapeutic potential of bnAbs.

## Abbreviations

AAV: adeno associated virus
bnAb: broadly neutralizing antibody
CD4bs: CD4 binding site
CDRH3: heavy chain complementarity determining region
Env: envelope glycoprotein
iGL: inferred germ line
mAb: monoclonal antibody
MPER: membrane proximal region
PNGS: predicted N-linked glycosylation site
UCA: unmutated common ancestor
V1/V2: variable loop 1 and 2
V3: variable loop 3
